# A divergent variant of *Grapevine leafroll-associated virus 3* is present in California

**DOI:** 10.1186/1743-422X-9-235

**Published:** 2012-10-13

**Authors:** YeeMey Seah, Abhineet M Sharma, Siming Zhang, Rodrigo PP Almeida, Siobain Duffy

**Affiliations:** 1 Department of Ecology, Evolution and Natural Resources, School of Environmental and Biological Sciences, Rutgers the State University of New Jersey, 14 College Farm Rd, New Brunswick, NJ, 08901, USA; 2 Department of Environmental Science, Policy and Management, University of California, Berkeley, CA, USA

**Keywords:** Ampelovirus, Wine, Mealybug

## Abstract

**Background:**

Grapevine leafroll-associated viruses are a problem for grape production globally. Symptoms are caused by a number of distinct viral species. During a survey of Napa Valley vineyards (California, USA), we found evidence of a new variant of *Grapevine leafroll-associated virus 3* (GLRaV-3). We isolated its genome from a symptomatic greenhouse-raised plant and fully sequenced it.

**Findings:**

In a maximum likelihood analysis of representative GLRaV-3 gene sequences, the isolate grouped most closely with a recently sequenced variant from South Africa and a partial sequence from New Zealand. These highly divergent GLRaV-3 variants have predicted proteins that are more than 10% divergent from other GLRaV-3 variants, and appear to be missing an open reading frame for the p6 protein.

**Conclusions:**

This divergent GLRaV-3 phylogroup is already present in grape-growing regions worldwide and is capable of causing symptoms of leafroll disease without the p6 protein.

## Findings

### Introduction

Grapevine leafroll disease (GLRD) is observed in all wine-making regions worldwide [1,2], limiting grape production by up to 40 percent [3]. Besides leaf rolling, other GLRD symptoms include abnormal pigmentation of the leaf interveinal area, disruption of the phloem and delayed grape maturation [3]. GLRD is caused by several related positive single-stranded RNA virus species in the family *Closteroviridae*, which contains the largest known plant RNA virus genomes [4]. All GLRD-causing viruses are phloem-limited [5] and infect *Vitis* hosts [6]. The mealybug-transmitted viruses are in the genus *Ampelovirus*, and *Grapevine leafroll-associated virus 2*, which has no known vector, is in the genus *Closterovirus*[6]. An additional GLRD-causing virus, *Grapevine leafroll-associated virus 7*, is still unclassified [7], although a recent proposal will place it in a new genus [8]. In fact, *Closteroviridae* recently underwent a taxonomic revision, and it is anticipated that the number of tentative GLRaV species will be reduced to five [8].

*Grapevine leafroll-associated virus 3* (GLRaV-3) is the type species of the genus *Ampelovirus*. Two distinct isolates, GP18 [9] and WA-MR, [10] have become representative of two major clades of GLRaV-3, but more intensive sampling revealed many genetically separated well-supported clades, potentially leading to seven subclades within GLRaV-3 [11]. The overall genomic diversity amongst GLRaV-3 had remained fairly limited [8] until the recent publication of a South African isolate (GH11), which had ~68% nucleotide identity with other GLRaV-3 variants [12], but showed higher identity to a partial sequence of GLRaV-3 from New Zealand (NZ-1).

During a recent survey of vineyards in Napa Valley, California USA, we found plants with divergent partial genome sequences of GLRaV-3, with close homology to NZ-1 (GLRaV-3e cluster) [11,13]. These plants were subsequently vegetatively propagated in our greenhouse at the University of California, Berkeley, and an isolate found in a symptomatic Merlot plant from Rutherford, California was selected to be fully sequenced. This plant was tested periodically for the presence of other GLRaV species by PCR of the coat protein-coding region from total nucleic acid (TNA) extractions as in [11]; no other GLRaV species was detected. Transmission experiments using the vine mealybug (*Planococcus ficus*, Hemiptera, Pseudococcidae) showed that this isolate is mealybug transmissible (Almeida, data not shown).

### Isolation and sequencing

RNA and TNA were purified as previously described [13]. TNA was purified for GLRaV detection and for sequencing all of the genome, except for the ends. The ends were sequenced using 3’ and 5’ RACE kits (Invitrogen, Carlsbad, CA) on purified RNA that was treated with a DNAse I, as suggested by the manufacturer. These and subsequent sequencing reactions were performed at the Barker Hall Sequencing Facility located on the U.C. Berkeley campus.

Sequencing of the full genome was performed using a primer walking strategy and reverse transcription was initiated outward from the coat protein-coding region. Forward primers (Table 1) were designed by aligning all available GLRaV-3 full genome sequences, including Napa Valley survey sequences where possible [13]. Virus-specific primers for reverse transcription were designed from sequencing data obtained above and to meet the manufacturer’s specifications of the Superscript II reverse transcriptase used in this study (Table 1). Four reverse transcription reactions were carried out per sample.

**Table 1:** Primers used in the amplification of the CA7246 genome, with locations referring to the 5' nucleotide, relative to CA7246's genome sequence

**Direction**	**Location**	**Name**	**Sequence(5’** → **3’)**	**Used for**
R	312	LR3E_FG300RACE	CAACACTACGCGCAAGAAAAGAGC	5' RACE
R	3254	LR3E_FG3258R	CGCTTGAAAGAACAGCCTGAAGATGTTC	RT, PCR
R	8193	LR3E_FG8194R	AGTGTCCATCCCATGGTAGAACAACCA	RT, PCR
R	11733	RT_FG11884	ACGTCTTTACGCACTTTCGAGAGA	RT, PCR
R	13357	LR3E-RDRP-R	AATTTCTCTGCGAGCTCAGGGCA	RT, PCR
R	14079	St E 13988-R	TACCACCGGTATGGTCGCCAGT	RT, PCR
R	14397	CP-580R	GCCCATAACCTTCTTACACA	RT, PCR
R	17805	St E 17713-R	CCCTCTTTCCACGACACACTTCG	RT, PCR
R	18443	LR3E_FG18376Rb	TATCACTATCGACTTTACGGACTAAT	RT, PCR
F	5	LR3E_FG5	ATGCTCTAGTAGGATTCGAACACGGCA	PCR
F	2973	LR3E_FG2976F	GTCGACAGGATCTATTCCTACGCGC	PCR
F	8045	LR3E_FG8045F	CACCTTTGTCGAACTACGTCACAGGG	PCR
F	10786	LR3E-RDRPFN-1	GGGGATAGCCGGATGTACACCGG	PCR
F	11335	LR3E-RDRPFN-2	TTTCGACGTCTCCTTCGTGAAG	PCR
F	13239	LR3E - Minus 710	TATGTACCAATCGAGTCGTTCG	PCR
F	13927	CP-130 F	GAACTGAAATTAGGGCAGATATA	PCR
F	14058	St E 13988-F	ACTGGCGACCATACCGGTGGTA	PCR
F	18044	3RACE_FG17991F	GCGATCGCTACTATAGTCGTGGTGA	3' RACE

Location refers to the 5’ nucleotide, relative to CA7246’s genome sequence, JQ796828. Reverse primers were used for both RT-PCR and PCR.

Primers for PCR were designed using conserved regions from the alignments above and with high melting temperatures to allow for a two-step PCR procedure using the Phusion Hot Start II Polymerase (Thermo-Fischer, Waltham, MA). Reverse transcription reactions from above were used as template. An initial two-minute, 98°C complete denaturation step was performed followed by 35 cycles of denaturing for 8 seconds at 98°C, followed by a joined primer annealing and extension step at 72°C for 30 seconds per kb of expected product. A final extension step for 7 minutes at 72°C was carried out to ensure complete extension of template. Amplicon sizes used to assemble the genome ranged between 3.5 kb and 8 kb, however, we were able to generate amplicons as large as 12 kb. A second round of PCR was carried out as above using the diluted 1^st^ PCR reactions as the template, amplifying with nested primers, and reducing the extension time to 20 seconds/kb. For each 1^st^ PCR sample, eight 2^nd^ PCRs were performed. All end products were visualized on a gel and then subsequently purified and concentrated using a kit (Zymo Research, Irvine, CA), and sent for sequencing. PCR products from the initial four or more RT-products were sequenced independently in both directions. The results were then manually checked and assembled using Vector NTI v.11 (Invitrogen). The assembly was then inserted into the alignment above and used to design new reverse transcription primers and reverse primers for PCR.

For both genomic ends, primers were designed using the sequencing data obtained above. For the 3’ end, poly-A tailing was performed prior to using the 3’ RACE Kit using a modified version of the manufacturer’s instructions to partially extend the ends (Ambion, Foster City, CA). Due to the appearance of multiple secondary products resulting from the lowered PCR specificity, the final product was treated with a T4 polymerase to blunt the 3’ overhangs for subsequent blunt cloning (New England Biolabs, Ipswich, MA). The product was cloned using a Zero Blunt Topo PCR cloning kit and Top10 chemically competent cells (Invitrogen). Colony PCRs and sequencing reactions were performed from 25 randomly chosen colonies using M13 primers. All colonies contained variable lengths of poly-A tailed product from the virus genome but only those with clean reads were utilized for assembly. For the 5’ end, the 5’ RACE kit instructions were followed. The PCR product was purified using a DNA Clean and Concentrator kit (Zymo Research) and sequenced.

### Sequence analysis

Annotation of the predicted open reading frames in the newly sequenced isolate, named CA7246 [GenBank: JQ796828], was done using MacVector (Cary, NC). ORFs were named according to sequence similarity and synteny with ORFs in GLRaV-3 [12]. Despite using an additional program (ORF Finder, http://www.ncbi.nlm.nih.gov/gorf/gorf.html) we could not find an ORF homologous to the GLRaV-3 ORF2 (encoding p6). The absence of this ORF was confirmed by sequencing of that region from additional five independent isolates. While this manuscript was in review, the sequence of GH11 [GenBank: JQ655295] was released, and was added to the analysis in revision. No ORF2 was detected in GH11 or the partial NZ-1 as well [12], indicating that p6 may not be an essential protein for GLRaV-3.

We then conducted a phylogenetic analysis on four important ORFs in GLRaV-3, and downloaded all available full-length GLRaV-3 RdRp, HSP70h, CP, and CPm sequences from GenBank on August 15, 2011 (GH11 was added in revision). The nucleotide sequences were manually aligned in Se-Al v2.0a11 (http://tree.bio.ed.ac.uk/software/seal/), appropriate nucleotide substitution models were then selected by ModelTest [14] based on Akaike’s Information Criterion, and used to infer maximum likelihood gene trees with 1000 bootstrap replicates in PAUP* v4.0beta [15].

**Figure 1:**
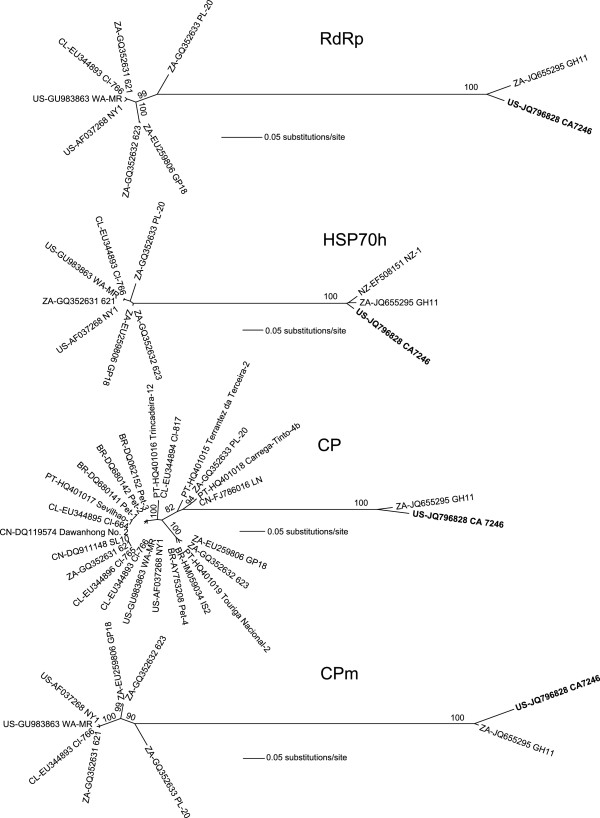
**Maximum likelihood trees constructed from full-length nucleotide sequences of*Grapevine leafroll-associated virus-3 (*GLRaV-3) RNA-dependent RNA polymerase(RdRp), heat shock protein-70 homolog(HSP70h), coat protein(CP)and minor, or diverged coat protein(CPm).** The GenBank accession numbers of the sequences are preceded by two-letter country codes identifying the location of isolation (BR=Brazil; CL=Chile; CN=China; NZ=New Zealand; PT=Portugal; US=United States; ZA=South Africa). The trees were subjected to 1000 bootstrap replicates; percent bootstrap supports of greater than 80 are reported at the nodes

These trees clearly show that CA7246 is more closely related to GH11 and the partial NZ-1 sequences than to other GLRaV-3 isolates (Figure 1). However, it is not known how these GLRaV-3 variants evolved to be so distinct from other GLRaV-3 strains. In order to assess whether any of the divergence of CA7246 was due to interspecific recombination, 200-base portions of the entire CA7246 genome were individually subjected to BLAST analysis to determine if any portion matched to any other taxa than GLRaV-3. The same analysis was conducted for the genome of GH11. All of these regions consistently showed homology to GLRaV-3 with no significant hits (BLAST score of ≥200) to other sequences in the non-redundant nucleotide collection in GenBank. The divergence of GH11/CA7246 from other GLRaV-3 variants appears to have arisen through mutation rather than recombination with any other characterized sequence.

The molecular weights of CA7246’s predicted protein products were calculated with the Sequence Manipulation Suite (http://www.bioinformatics.org/sms2/) [16] and are given in Table 2. Several of the GLRaV-3 proteins are named for their inferred protein molecular weights, and two of CA7246’s homologues differed in molecular weight: 19.4 kDa and 6.2 kDa for the “p19.6”, and the “p7” proteins, respectively.

**Table 2:** **Percent amino acid and nucleotide identities between the untranslated regions and protein**-**coding genes (non-gapped columns) of CA7246 and isolates GH11, GP18, WA**-**MR and the partially sequenced isolate NZ**-**1**

**Gene**	**ORF**	**Length**	**Mass**	**% amino acid identity**				**% nucleotide identity**			
		(**nt**)	(**kDa**)	**GH11**	**GP18**	**WA**-**MR**	**NZ**-**1**	**GH11**	**GP18**	**WA**-**MR**	**NZ**-**1**
5’ UTR	--	737	--	--	--	--	--	81.8	48.9	50.8	--
MET/HEL	1a	6714	246.81	91.8	71.0	71.0	--	89.5	66.0	66.0	--
RdRp	1b	1629	62.05	97.8	88.0	89.6	--	93.8	77.5	78.5	--
p5	3	138	5.14	93.3	77.8	75.6	93.3	92.8	70.3	72.5	93.5
HSP70h	4	1650	59.26	96.2	85.4	86.0	95.5	93.0	75.3	75.1	91.9
p55	5	1452	55.06	94.4	75.0	73.9	94.0	91.8	68.8	68.2	88.6
CP	6	942	34.63	96.2	88.2	90.7	--	92.1	77.7	79.2	--
CPm	7	1434	53.02	93.1	77.8	77.8	--	90.9	71.6	71.6	--
p21	8	558	21.39	93.5	77.8	77.3	--	91.2	74.7	74.2	--
p19.6	9	534	19.44	91.0	54.2	56.5	--	90.5	60.7	62.4	--
p19.7	10	540	19.70	86.6	63.1	61.5	--	86.3	64.4	63.7	--
p4	11	111	3.95	77.8	30.6	25.0	--	83.8	44.1	39.6	--
p7	12	183	6.24	90.7	61.1	61.1	--	91.5	64.2	62.4	--
3’ UTR	--	256	--	--	--	--	--	96.6	78.8	79.9	--

The percent identities between sequences with gapped alignments were calculated using only the common non-gap columns. Protein names and ORF numbering are as in the type sequence of GLRaV-3, though ORF2 (and its product, p6), do not appear in CA7246 or GH11.

The predicted ORFs and untranslated regions from CA7246 were also aligned and compared to three other GLRaV-3 complete sequences (Table 2): to GH11 [GenBank: JQ655295], WA-MR [GenBank: GU983863] and GP18 [GenBank: EU259806], and to the partial sequence of NZ-1 [GenBank: EF508151]. Nucleic and amino acid percent identities between CA7246 and the four GLRaV-3 sequences were calculated using the Percent Identity tool in UCSF Chimera’s MultAlign Viewer [17]. These ORF-by-ORF comparisons show that CA7246 and GH11 are more closely related than they are to other GLRaV-3 variants across their genomes.

However, the CA7246 genome is 9.6% divergent from GH11 by nucleotide sequence, indicating they did not recently diverge from one another. Their 3’UTRs were more identical than their 5’UTRs, which is consistent with the wider diversity of 5’UTR structures that are observed among GLRaV-3 isolates [10,18]. The amino acid identities of their predicted protein products were higher, with the notable exception of p4, which was only 77.8% identical (Table 2). p4 was also the site of the greatest difference between GH11/CA7246 and the other GLRaV-3 variants, with at most 30.6% amino acid identity (Table 2). This bolsters our previous observation of completely neutral evolution in this ORF [13], and further suggests that this annotated ORF may not be translated, or that it may have a non-essential function.

Isolates of a new phylogroup of GLRaV-3 are present on three continents, and their sequences have diverged sufficiently that it is clear that these isolates dispersed from one another some time ago. We suspect this divergent GLRaV-3 variant has a wide geographic range, and may already be present in other wine-growing regions.

## Abbreviations

RdRp: RNA-dependent RNA polymerase; HSP70h: Heat shock protein 70 homolog; CP: Coat protein; CPm: Minor coat protein.

## Competing interests

The authors declare no competing interests.

## Authors’ contributions

AMS isolated and sequenced the virus. SZ sequenced and assembled the viral sequence. YMS conducted the phylogenetic analysis. RPPA, SD and YMS wrote the manuscript. All authors read and approved the final manuscript.
